# Supplementary material for: High-power transient 12–30 Hz beta event features as early biomarkers of Alzheimer’s disease conversion: An MEG study
Source: Imaging Neurosci (Camb). 2025 Jul 14;3:IMAG.a.69. doi: 10.1162/IMAG.a.69 (PMC12330844; doi:10.1162/IMAG.a.69)
Supplement: Supplementary Material [file IMAG.a.69_supp.pdf]

Supplementary Material

"Mirror" events

The MEG activity of medial regions is characterized by the presence of synchronized, contralateral, spurious mirror events (see TFRs in Figure S1). This occasional phenomenon is caused by the limited resolution of MEG/volume conduction. When using the MNE dipole-constrained source reconstruction method, these duplicated events are noticeable because they are characterized by a mutual inversion of the time series. We performed a removal step of duplicated mirror events: when two detected events shared the same (but contralateral) ROI, epoch, time and frequency, we discarded the one with lower amplitude.

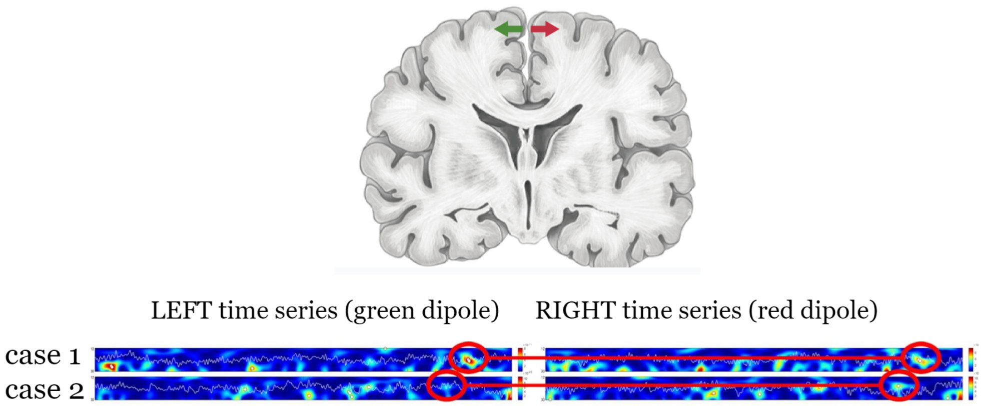

Figure S1: Example of mirror event in left ACC and right ACC due to spatial smearing in source reconstruction. The events considered at the end of the detection process were the left one in the case1, and the right one in the case2, due to their amplitudes.

Correction of TFR by removing the 1/f aperiodic component

To test the effect of 1/f effect in the detection of high-power spectral events, we removed the aperiodic component from TFRs. First, we calculated the aperiodic component by applying the FOOOF algorithm to the power spectral density (PSD) extracted from all TFRs for each subject. Next, we computed a normalization factor following the PAPTO method (see Figure 3a) (Brady & Bardouille, 2022) (refer to find PAPTO bursts, lines 201–205).

The normalization factor was defined as:

$$nc = \frac{10^{\mathsf{Subj}_k\_\mathsf{offset}}}{fVec^{\mathsf{Subj}_k\_\mathsf{exponent}}}$$

Using this factor, we normalized each TFR trial as follows (see Figures 3b and 3c):

$$TFR_{\mathsf{foofed}} = \frac{TFR}{nc}$$

After normalization, we detected spectral events in the TFR fooofed signal that exceeded the 6× Factor-of-the-Median (FOM) threshold (see Figures S2b and S2d). Figure 3d illustrates the TFR amplitude at each frequency step, comparing the 6×FOM threshold with a fixed 6×nc threshold. Finally, in Figure 3e, we compare the number of detected events (using the 6×FOM threshold) in the original TFR versus the TFR fooofed, before and after the removal of the 1/f aperiodic component.

Correction of TFR by removing the 1/f aperiodic component does not affect the elemental features calculated by SpectralEvents. The number of events detected before and after the 1/f correction is identical, as the difference (post - pre) is centered around zero for most trials (Figure S2e). Specifically, the number of events detected in the beta [12–30] Hz range before and after aperiodic removal is not statistically different (Wilcoxon signed-rank test: $W = 7.2 \times 10^6$, $p = 0.961$). Furthermore, the increment (post - pre) is not statistically different between the CONV and NOCONV groups (Mann-Whitney $U = 2.8 \times 10^7$, $p = 0.255$).

For the alpha [5–10] Hz range, while the number of events detected is higher post-aperiodic removal ($W = 4.3 \times 10^5$, $p < 0.001$), this increase in the number of alpha events detected was equally distributed between the CONV and NOCONV groups ($U = 2.8 \times 10^7$, $p = 0.268$).

Furthermore, similar results were obtained when analyzing the effect of aperiodic component removal on other spectral event features (see Supplementary Figure 3). We attribute this to the fact that the $6 \times$ FOM threshold dynamically adjusts by frequency, targeting high-power bursts of periodic activity while adapting to 1/f noise at each frequency step.

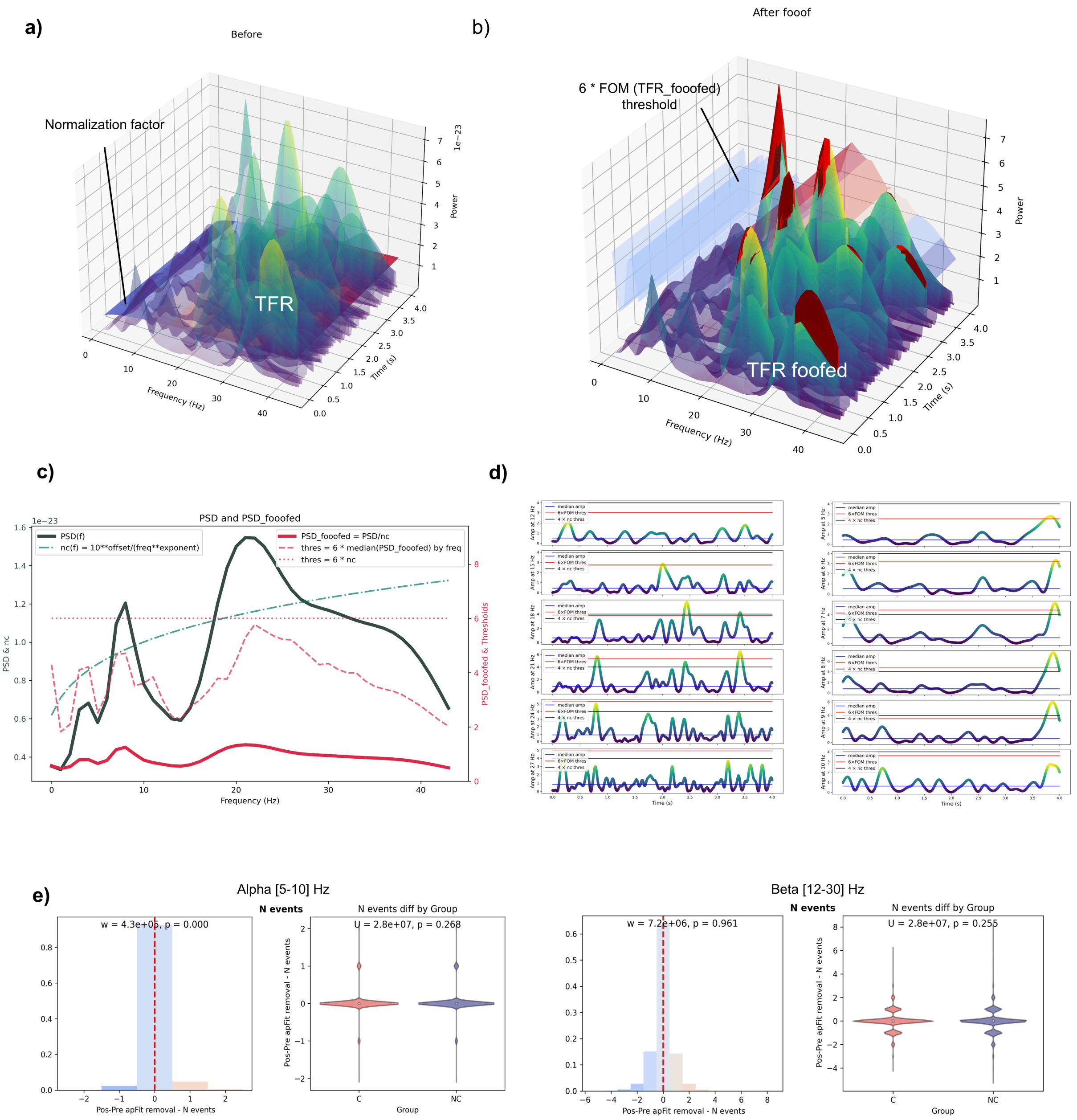

Figure S2: Correction of TFR by removing the 1/f aperiodic component. a) Time-frequency representation (TFR) and normalization factor (nc) for one trial. b) TFR after applying the FOOOF-based correction, where high-power spectral events are detected above the 6× FOM threshold. c) Power spectral density before and after normalization (PSD fooofed) for one trial alongside 6×FOM and 6×nc thresholds. d) Time-domain TFR fooofed amplitude by frequency steps with 6×FOM and 6×nc thresholds. e) Statistical comparison of the number of detected spectral events in alpha [5–10] Hz and beta [12–30] Hz bands before and after 1/f correction, including group-level differences.

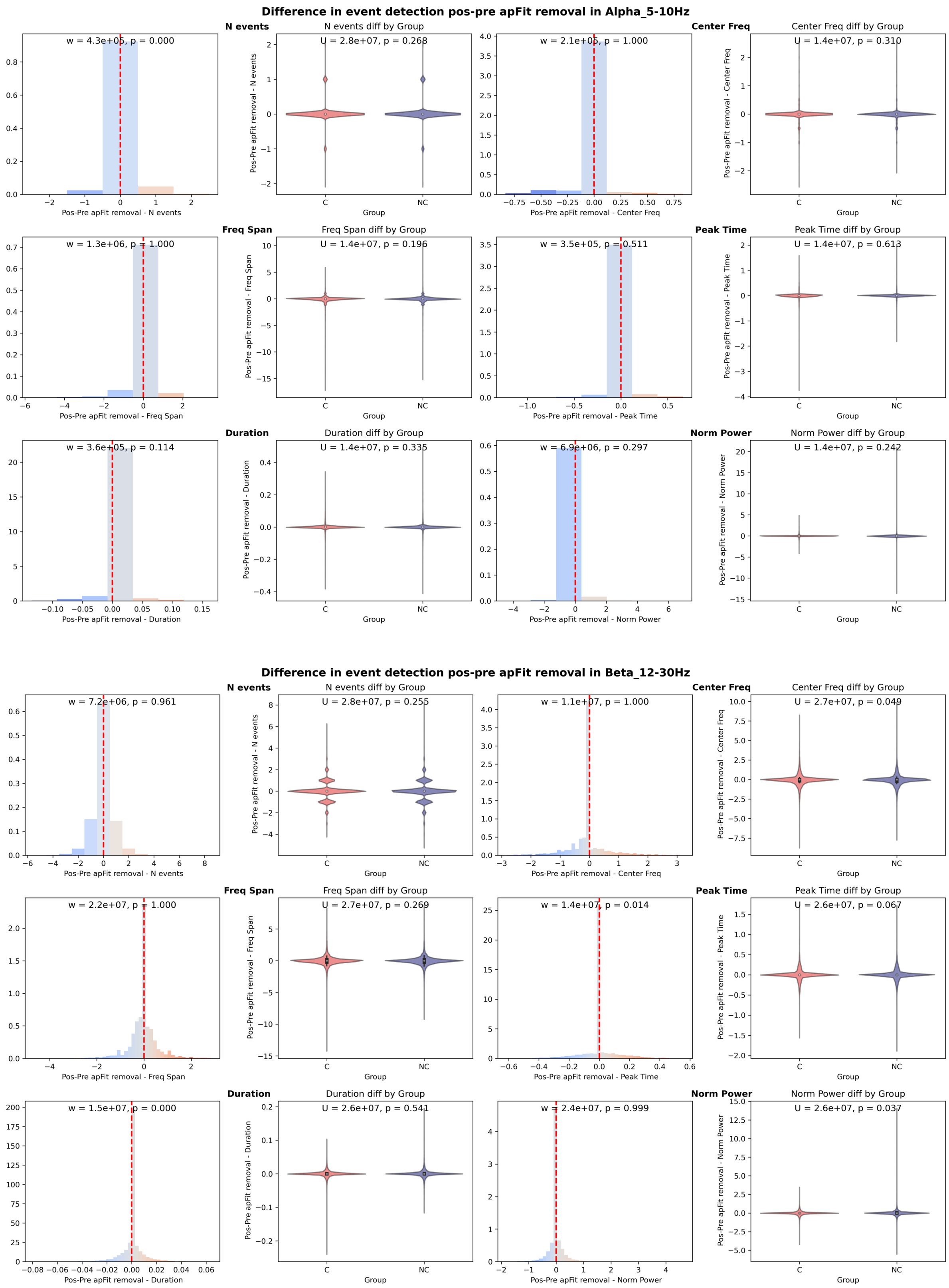

Figure S3: Differences in spectral event features (pos-pre) aperiodic component removal by group: aperiodic offset, aperiodic exponent, goodness of fit (R^2), periodic peaks—center frequencies, periodic peaks—power, and periodic peaks—bandwidth.

Event threshold analysis

A

B

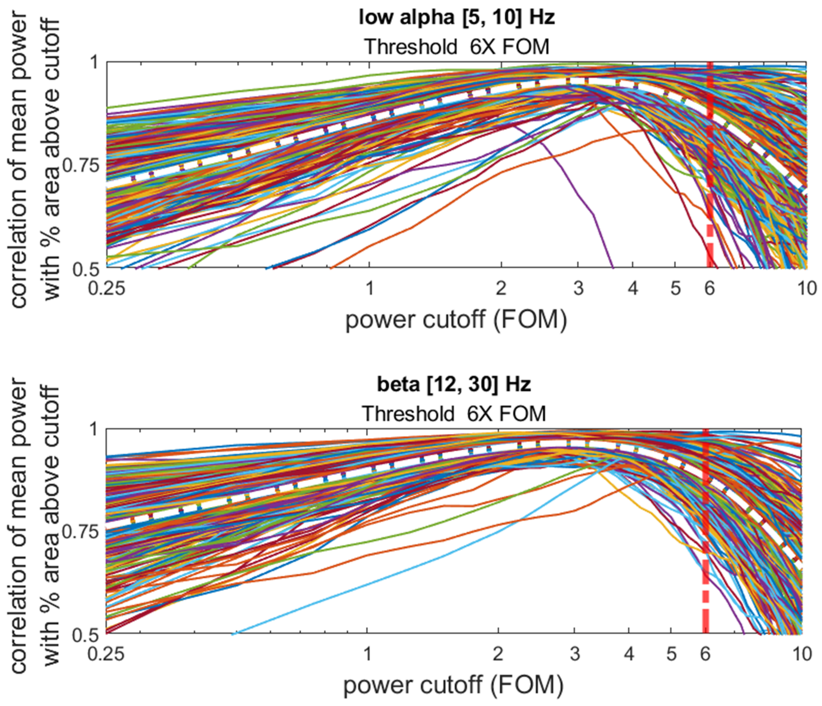

Figure S4: Average relationship between power cutoff FOM and correlation of mean power with percentage of area above cutoff. a) Analysis over events selected in alpha frequency [5-10] Hz and visualisation of 6$\times$ threshold (red line). b) Analysis over events selected in beta frequency [12-30] Hz and visualisation of 6$\times$ threshold (red line).

Freq.	ROI	Feature	t-Stat	p-value	BH adjusted p-value	Cohen d
Low alpha [5-10] Hz	ACC	Ev. Rate	1,705	0,045	0,18	-0,226
		Duration	-2,084	0,981	0,993	0,258
		Fspan	1,484	0,07	0,203	-0,126
		Pow.FOM	-1,11	0,866	0,993	0,078
	PC	Ev. Rate	1,113	0,134	0,267	-0,188
		Duration	-2,507	0,993	0,993	0,376
		Fspan	-1,453	0,926	0,993	0,267
		Pow.FOM	-1,221	0,888	0,993	0,16
Beta [12-30] Hz	ACC	Ev. Rate	1,716	0,956	0,993	-0,217
		Duration	-2,67	0,004	0,033 **	0,383
		Fspan	1,241	0,892	0,993	-0,086
		Pow.FOM	-1,438	0,076	0,203	0,224
	PC	Ev. Rate	-1,183	0,119	0,267	0,194
		Duration	-2,37	0,009	0,05 *	0,336
		Fspan	2,213	0,986	0,993	-0,358
		Pow.FOM	-3,744	0	0,002 **	0,561

Table S1: Statistical comparison for x4 FOM of event features mean averaged for CONV and NOCONV groups in low-alpha [5-10] Hz and beta [12-30] Hz frequency bands. Significant differences ($p < 0.05$) are marked with the asterisk (**). Statistical tendency ($p < 0.1$) is marked with the asterisk (*).

**Freq.**	**ROI**	**Feature**	**t-Stat**	**p-value**	**BH adjusted p-value**	**Cohen d**
Low alpha [5-10] Hz	ACC	Ev. Rate	-0,497	0,69	0,91	-0,077
		Duration	0,679	0,249	0,639	-0,247
		Fspan	0,082	0,467	0,91	0,037
		Pow.FOM	-0,33	0,629	0,91	-0,029
	PC	Ev. Rate	-0,585	0,72	0,91	0,078
		Duration	-0,959	0,831	0,91	0,047
		Fspan	-1,052	0,853	0,91	0,164
		Pow.FOM	-0,804	0,789	0,91	0,162
Beta [12-30] Hz	ACC	Ev. Rate	-0,585	0,28	0,639	0,095
		Duration	-1,831	0,034	0,138	0,287
		Fspan	0,295	0,616	0,91	-0,007
		Pow.FOM	-0,973	0,166	0,531	0,083
	PC	Ev. Rate	-3,446	0	0,003 **	0,466
		Duration	-2,221	0,014	0,074 *	0,317
		Fspan	1,721	0,956	0,956	-0,22
		Pow.FOM	-3,602	0	0,003 **	0,498

Table S2: Statistical comparison for x8 FOM of event features mean averaged for CONV and NOCONV groups in low-alpha [5-10] Hz and beta [12-30] Hz frequency bands. Significant differences ($p < 0.05$) are marked with the asterisk (**). Statistical tendency ($p < 0.1$) is marked with the asterisk (*).

Probability density plots of event features

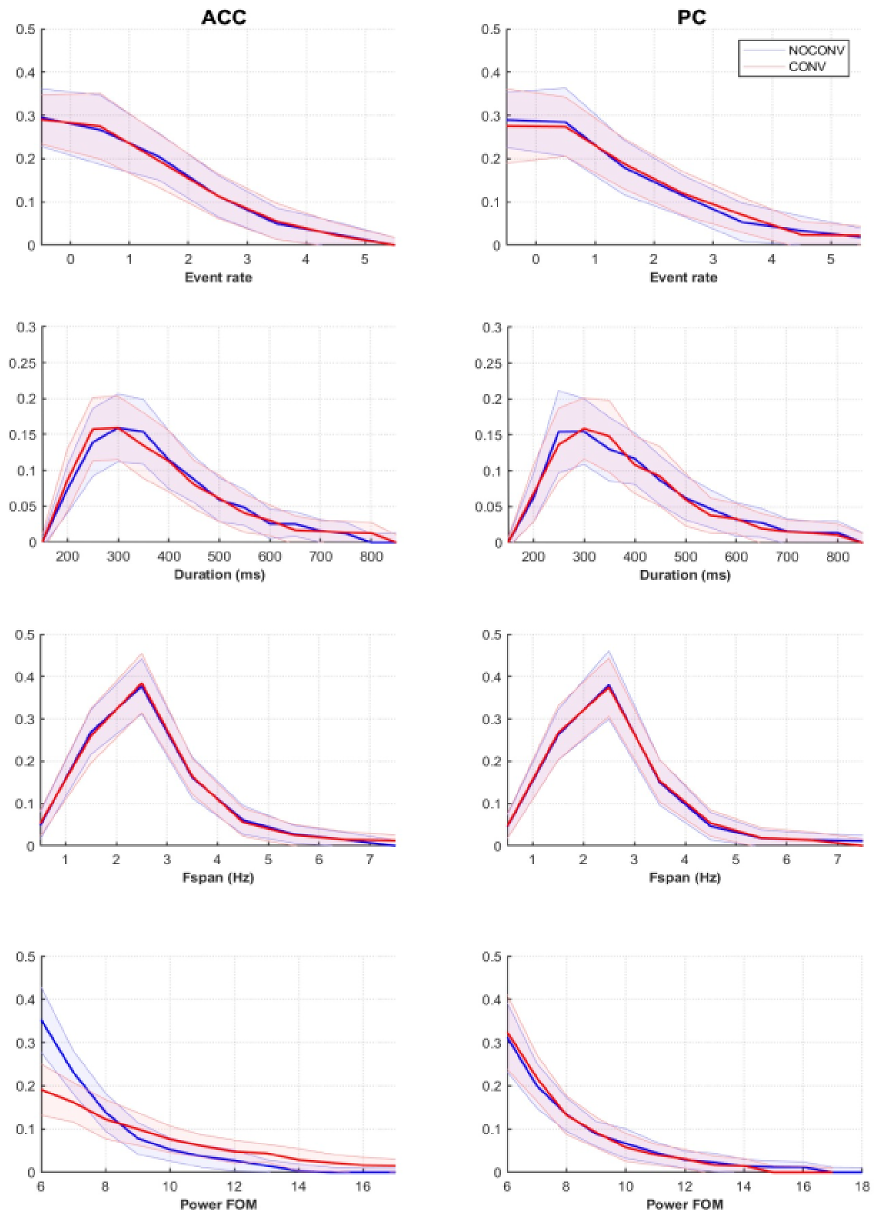

Figure S5: Probability density plots for each event features in low-alpha [5-10] Hz band for 4 seconds trials.

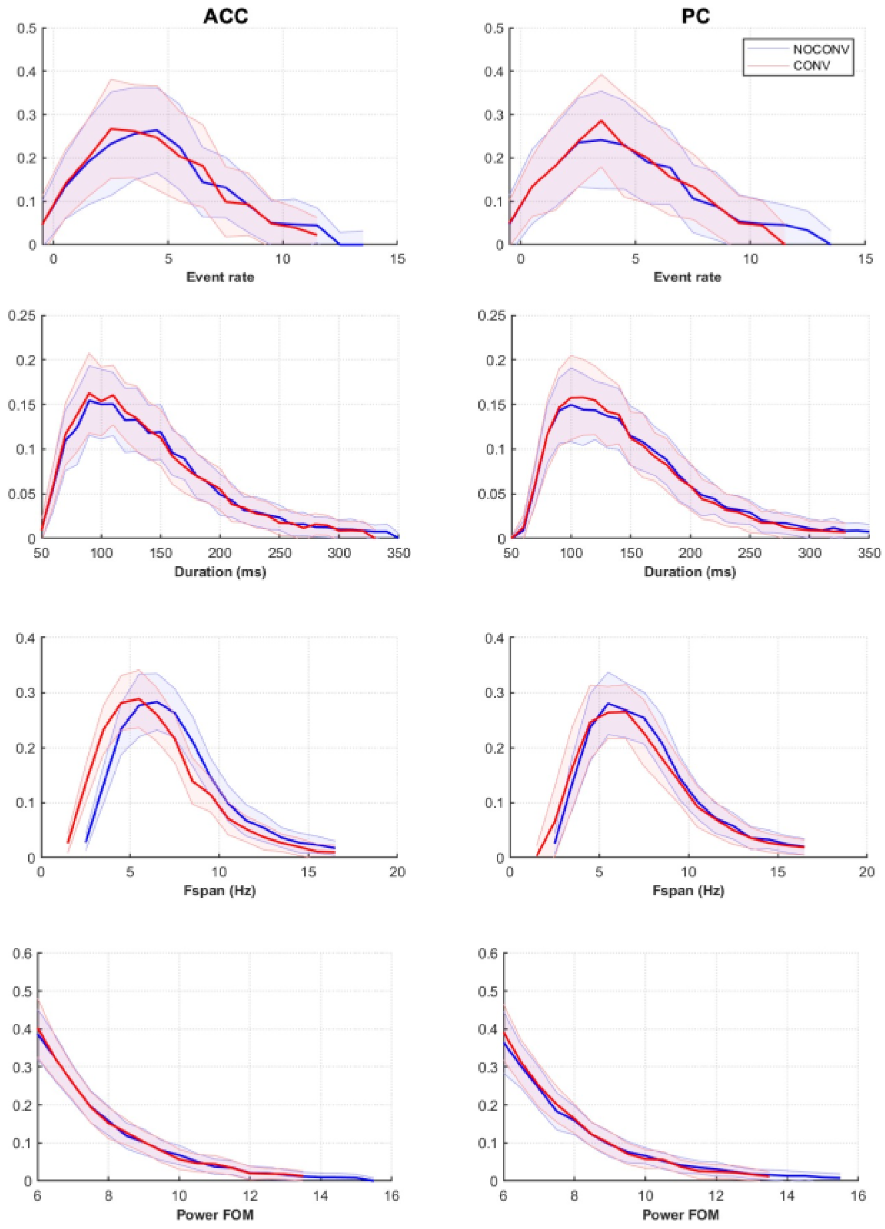

Figure S6: Probability density plots for each event features in beta [12-30] Hz band for 4 seconds trials.

Comparison of beta event peak frequency between AD CONV and NOCONV group

Although peak frequency was not included in the main characterization of transient events to maintain coherence with the approach used in previous work (Shin et al., 2017), we conducted additional analyses to explore potential group differences, given its relevance to oscillatory slowing in Alzheimer's disease.

No statistically significant differences in peak frequency were found between groups in either region. In the alpha band, differences were non-significant in both ACC ($t(167) = 0.09$, $p = .534$) and PC ($t(161) = -1.31$, $p = .097$). Similarly, in the beta band, no significant differences were observed in ACC ($t(168) = -0.22$, $p = .412$) or PC ($t(167) = 1.67$, $p = .951$).

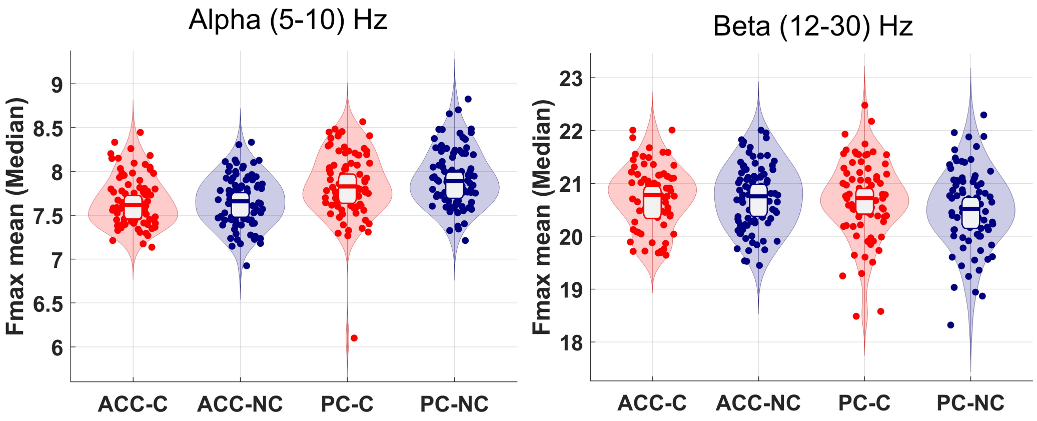

Figure S7: Comparison of beta event peak frequency between AD CONV and NOCONV group
